# Protein interaction network topology uncovers melanogenesis regulatory network components within functional genomics datasets

**DOI:** 10.1186/1752-0509-4-84

**Published:** 2010-06-15

**Authors:** Hsiang Ho, Tijana Milenković, Vesna Memišević, Jayavani Aruri, Nataša Pržulj, Anand K Ganesan

**Affiliations:** 1 Department of Biological Chemistry, University of California, Irvine, CA 92697-1700, USA; 2 Department of Computer Science, University of California, Irvine, CA 92697-3435, USA; 3 Department of Dermatology, University of California, Irvine, CA 92697-2400, USA; 4 Department of Computing, Imperial College London SW7 2AZ, UK

## Abstract

**Background:**

RNA-mediated interference (RNAi)-based functional genomics is a systems-level approach to identify novel genes that control biological phenotypes. Existing computational approaches can identify individual genes from RNAi datasets that regulate a given biological process. However, currently available methods cannot identify which RNAi screen "hits" are novel components of well-characterized biological pathways known to regulate the interrogated phenotype. In this study, we describe a method to identify genes from RNAi datasets that are novel components of known biological pathways. We experimentally validate our approach in the context of a recently completed RNAi screen to identify novel regulators of melanogenesis.

**Results:**

In this study, we utilize a PPI network topology-based approach to identify targets within our RNAi dataset that may be components of known melanogenesis regulatory pathways. Our computational approach identifies a set of screen targets that cluster topologically in a human PPI network with the known pigment regulator Endothelin receptor type B (EDNRB). Validation studies reveal that these genes impact pigment production and EDNRB signaling in pigmented melanoma cells (MNT-1) and normal melanocytes.

**Conclusions:**

We present an approach that identifies novel components of well-characterized biological pathways from functional genomics datasets that could not have been identified by existing statistical and computational approaches.

## Background

Identifying the complete set of genes that regulate a biological phenotype is a challenge of systems biology. Availability of systems-level protein-protein interaction (PPI), gene expression, and functional genomics (FG) data has facilitated the development of integrative computational approaches to uncover genes involved in biological processes [1]. Integration of *C. elegans *FG data [1] with existing gene expression and PPI data has facilitated the discovery of co-expressed gene networks [2], early embryogenesis control networks [3], and large-scale protein function networks [4]. Integrating *Drosophila *RNAi datasets with PPI networks helped identify novel functional regulators of biological phenotypes, demonstrating that PPI networks and RNAi datasets can be effectively integrated to derive additional functional information from RNAi screens [5]. Application of these methods to mammalian RNAi datasets has been more problematic secondary to higher false positive and false negative rates of mammalian RNAi screens [6]. Biological pathways are distinct, experimentally-validated subnetworks of proteins within the larger PPI network that interact with each other by well defined mechanisms to regulate a specific biologic phenotype. While currently available methods can identify components of RNAi datasets that interact with each other within PPI networks [7], no method currently exists to determine which of these screen "hits" are novel components of well defined pathways known to regulate the process under study.

Numerous studies have identified molecular determinants of pigment variation: 127 mouse coat color genes have been identified [8] that coordinately regulate the transcription, translation, and intracellular trafficking of melanogenic enzymes [9]. These studies have identified the master regulator of melanocyte transcription microphthalmia-associated transcription factor (MITF) [10], several melanogenic enzymes [9], and regulators of melanosome formation and trafficking [11]. Despite these advances, our current understanding of skin and eye color variability is incomplete [12].

Recently, we utilized a systems-level FG platform to identify 92 novel genes that regulate melanin production, novel regulators of melanin secretion, and novel depigmenting agents [13]. Notably, our approach failed to identify many known regulators of melanogenesis among our top tier hits, and annotation data failed to identify connections between many screen targets and biological pathways known to regulate melanogenesis. In this study, we apply PPI network topology-based computational methods to identify genes within our FG dataset that are novel components of biological pathways known to regulate melanogenesis.

## Results and Discussion

### Topological similarity uncovers novel melanogenesis-related regulatory network members within a functional genomics dataset

**Figure 1:**
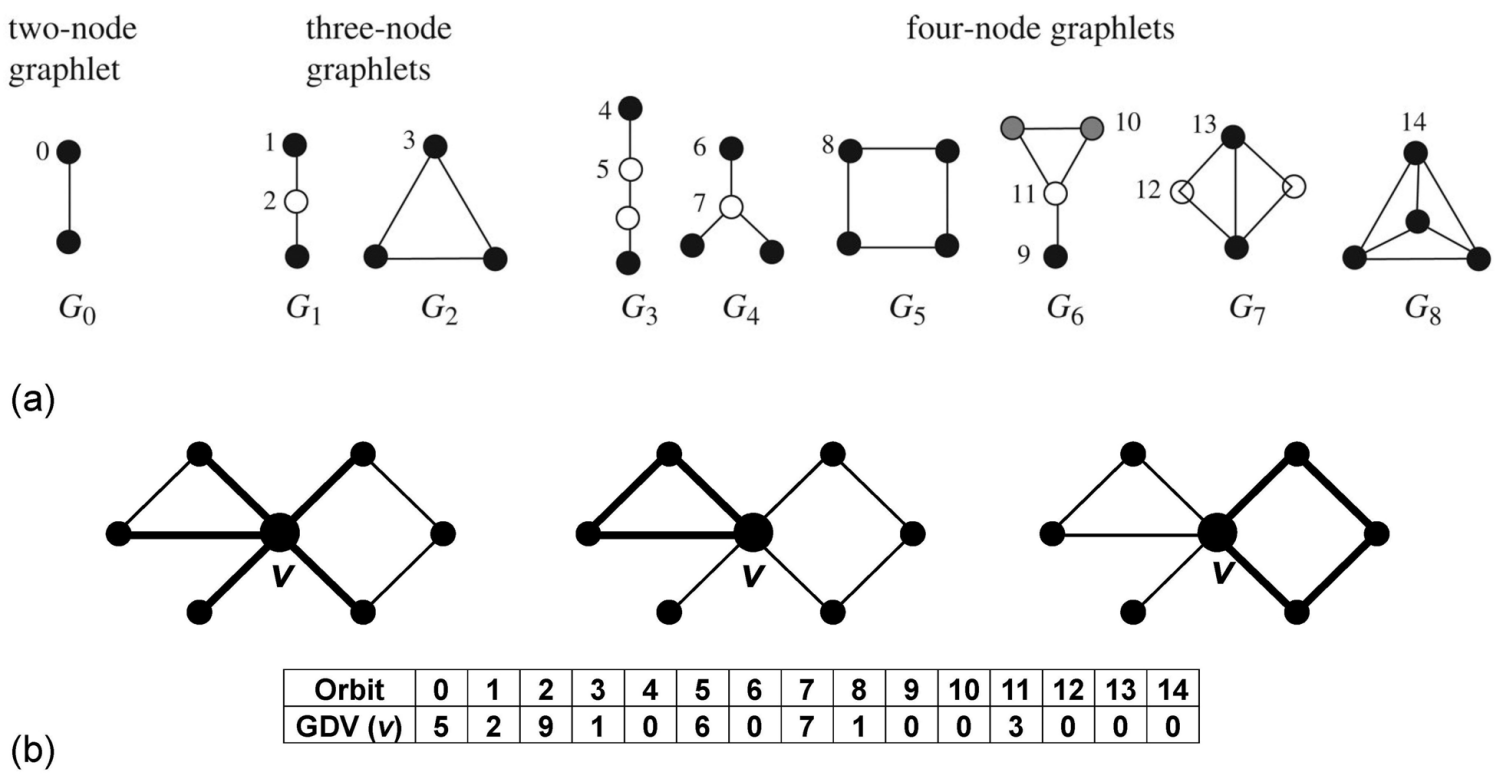
**Graphlet degree vectors**. (**a**) All 9 graphlets on 2, 3 and 4 nodes, denoted by G_0_, G_1_, ..., G_8_; they contain 15 topologically unique node types, called "automorphism orbits," denoted by 0, 1, 2 ..., 14. In a particular graphlet, nodes belonging to the same orbit are of the same shade [52]. (**b**) An illustration of the "Graphlet Degree Vector" (GDV), or a "signature" of node *v*; coordinates of a GDV count how many times a node is touched by a particular automorphism orbit, such as an edge (the leftmost panel), a triangle (the middle panel), or a square (the rightmost panel). Hence, the degree is generalized to a GDV [17]. The GDV of node v is presented in the table for orbits 0 to 14: v is touched by 5 edges (orbit 0), end-nodes of 2 graphlets G_1 _(orbit 1), etc. Values of the 73 coordinates (for all of the 30 2-5-node graphlets) of the GDV of node v are presented in Additional file 1 Figure S1.

In this study, we examine the interrelationship between PPI network topology and both known and newly identified biological pathways that regulate melanogenesis. In PPI networks, nodes correspond to proteins and edges represent possible interactions amongst them. To increase the coverage of PPIs, we analyze the union of the *physical *human PPI networks from HPRD [14], BioGRID [15], and by Radivojac et al. [16], consisting of 47,303 interactions amongst 10,282 proteins. We characterize the topology of nodes' neighborhoods in the PPI network with their "graphlet degree vectors" (GDVs) [17]; graphlets are small connected induced subgraphs of a network (Figure 1). GDV of a node, also called the node "signature," generalizes the degree of a node that counts how many edges the node touches into the vector of graphlet degrees that counts how many graphlets of a given type, such as a triangle or a square, the node touches (see Figure 1 and additional file 1 Figure S1 for an illustration). All 2-5-node graphlets, presented in Figure 1 and additional file 1 Figure S1, are taken into account. Clearly, the degree of a node is the first coordinate in this vector, since an edge (graphlet G_0 _in Figure 1) is the only 2-node graphlet. To take into account the symmetry groups within a graphlet, the notion of automorphism orbits (or just orbits, for brevity) is used for all graphlets with 2-5-nodes. For example, it is topologically relevant to distinguish between nodes touching a 3-node linear path (graphlet G_1 _in Figure 1) at an end, or at the middle node. By taking into account these symmetries between nodes of a graphlet, there are 73 different orbits for 2-5-node graphlets, numbered from 0 to 72 in Figure 1 and additional file 1 Figure S1. Thus, GDV of a node, describing the complex topological wiring of node's up to "4-deep neighborhood," has 73 coordinates [17]. We compute pairwise "similarities" between GDVs; higher signature similarity corresponds to higher topological similarity of the nodes' "network surroundings" (see [17] for details). We say that two proteins are "signature-similar" if their GDV similarity is above a given threshold. This measure has been used to demonstrate that topologically similar proteins have similar functions [17,18].

To find proteins topologically similar to known melanogenesis regulators in the context of the PPI network, we compute GDVs for pigment regulators identified in our siRNA screen (henceforth called Screen Pigment Regulators (SPRs)). As previous studies demonstrated that many known pigment regulators (KPRs) were not present in our top tier targets but did have some impacts on pigment production [13], we define SPRs as genes whose siRNAs have some impact on pigment production (Z-score < -1 or >1) (Additional File 2 Table S1). 1,244 SPRs are present in the existing PPI network. Also, the existing PPI network contains 41 KPRs from the ESPCR database, 10 of which are SPRs at the same time [8]. We note that a choice of a higher Z-score threshold might be more appropriate; however, such a choice omitted several KPRs from the analyzed dataset and resulted in a limited number of SPRs in the dataset that was insufficient for any statistical analysis.

**Figure 2:**
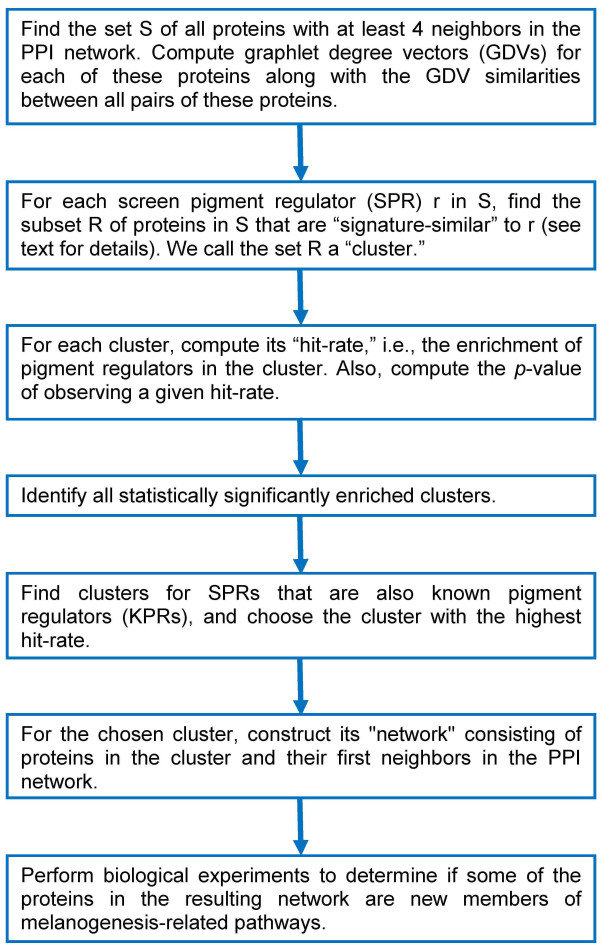
**Application of protein interaction network topology to uncover novel melanogenesis gene networks from functional genomics data**. Step-by-step methodology diagram is presented.

To identify specific melanogenesis regulatory subnetworks hidden in our FG dataset, we identify SPRs with similar GDVs in the PPI network, with the hypothesis that some signature-similar SPRs would be components of the same protein subnetwork. For each SPR, we form a "cluster" containing that protein and all other proteins in the network that are signature-similar to it (see Methods). Thus, the clusters contain proteins that have similar topologies within the PPI network. We measure the statistical significance of SPRs to cluster together. We denote by "hit-rate" the percentage of proteins in a cluster that are SPRs, excluding the protein for which the cluster was formed (see Methods and Figure 2). We find that 26 of our clusters are statistically significantly enriched with SPRs at p-value threshold of SPR-enrichment of 0.05 (see Methods); the list of proteins for which the clusters were formed, together with their hit-rates and p-values, is provided in additional file 3 Table S2. By analyzing random clusters (see Methods) and by using the same enrichment and statistical significance criteria, we find that it is unlikely to observe 26 statistically significantly SPR-enriched clusters by chance (p-value = 0.009; see Methods). Note that the relatively low number of statistically significantly SPR-enriched clusters is expected due to incompleteness of SPR (KPR) data set. Since the number of SPRs (KPRs) will increase in the future, it is expected that the enrichment of each of the clusters will increase and their p-values will decrease. Consequently, the number of statistically significantly enriched clusters will increase as well.

**Figure 3:**
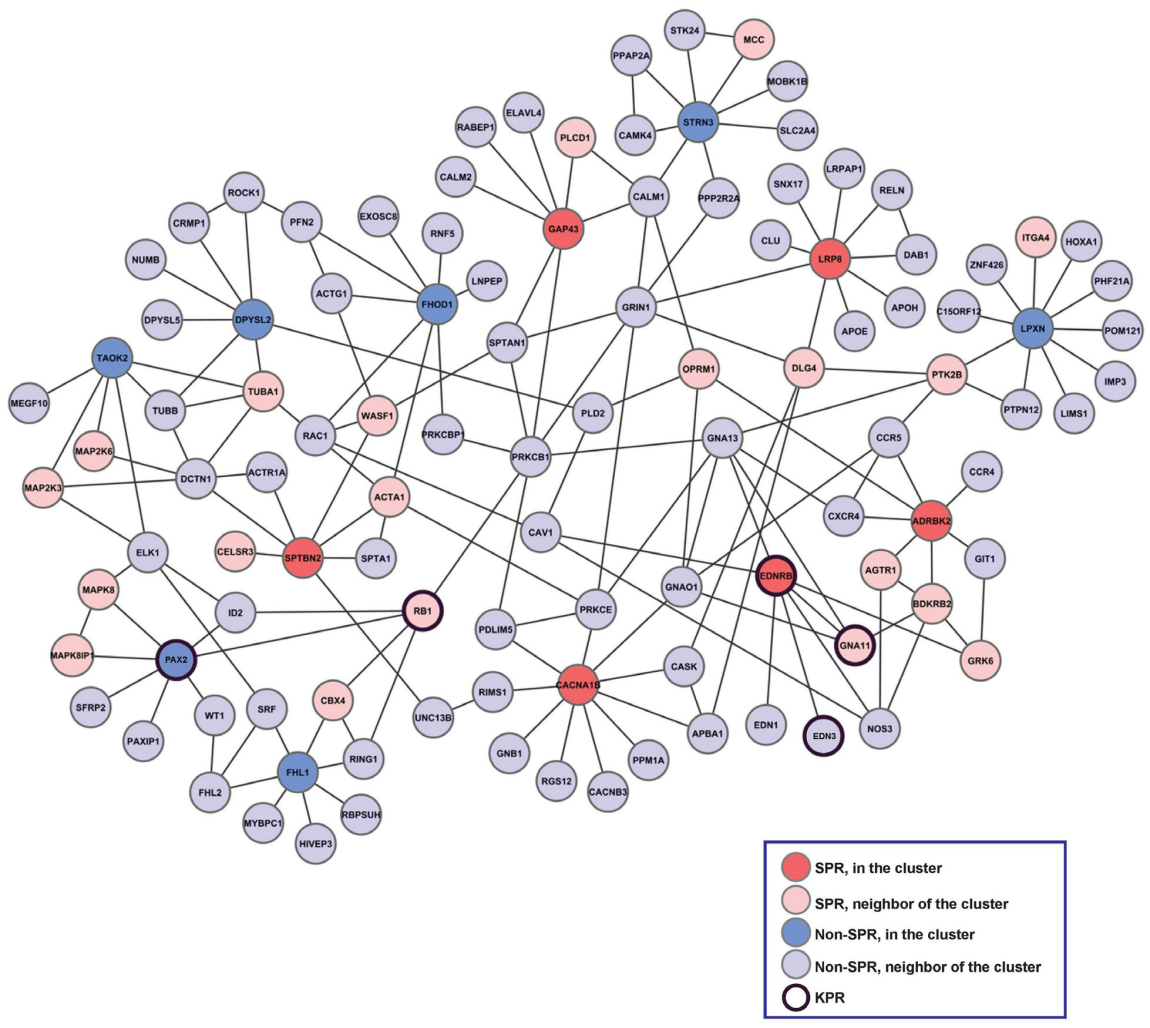
**EDNRB network containing proteins in the EDNRB cluster and their direct neighbors, as well as all interactions from the human PPI network that exist between these nodes**. Known pigment regulators (KPRs) from the ESPCR database and screen pigment regulators (SPRs) are noted.

By uncovering clusters that are statistically significantly enriched with SPRs, we demonstrate that genes with functional roles in melanogenesis indeed have similar topological signatures in the PPI network. To determine whether any of the SPRs are novel components of *known *melanogenesis regulatory pathways, we examine clusters of 10 SPRs that are at the same time KPRs and choose for further analyses the cluster with the highest hit-rate. The identified cluster is the cluster formed for EDNRB. It contains 13 proteins in total and its hit-rate is 41.67%. We analyze this cluster despite that it is not statistically significantly SPR-enriched since (1) its hit-rate could be considered "state of the art" given the noisiness of biological experiments, SPR (KPR) data set, the PPI network data that we use [18], and (2) statistically non-significant results may be biologically and scientifically interesting and important, whereas statistically significant results can turn out not to be [19]. The fact that five SPRs topologically cluster with EDNRB (which is both an SPR and a KPR) and given that we have previously demonstrated the link between topological and functional similarity [17,18,20], we conclude that our FG dataset may contain unappreciated members of biological pathways or subnetworks that regulate melanogenesis. Thus, to uncover EDNRB's corresponding melanogenesis-related pathway, we form the "EDNRB network" by expanding in the PPI network around the proteins in EDNRB cluster as follows. The "EDNRB network" (Figure 3) contains the set of proteins in EDNRB cluster and their direct neighbors, along with all interactions from the human PPI network that exist between these nodes. There are 109 nodes in EDNRB network (Figure 3): the proteins that are in the cluster part of the network (i.e., are topologically similar to EDNRB) include 2 KPRs (EDNRB, PAX2),5 SPRs (ADRBK2, CACNA1B, GAP43, LRP8, and SPTBN2) and 6 proteins not detected by our siRNA screen (TAOK2, DPYSL2, FHOD1, LPXN, FHL1, and STRN3); the proteins that are the neighbors of the cluster proteins in the EDNRB network include 3 KPRs (EDN3, GNA11, and RB1), 18 SPRs (BDKRB2, AGTR1, GRK6, PTK2B, ITGA4, DLG4, OPRM1, PLCD1, MCC, WASF1, ACTA1, TUBA1, CELSR3, CBX4, MAPK8, MAPK8IP1, MAP2K3, MAP2K6), and 75 proteins that are not detected by our siRNA screen. We include direct neighbors of the cluster proteins because the network without them is disconnected. Also, since biological pathways are contiguous "linear" structures in PPI networks, including them will aid in detecting new members of a biological pathway.

An alternative approach to finding new pathway members would be to consider simply an SPR and its direct neighbors as cluster members, since it has been shown that proteins that are closer in the network (i.e., direct neighbors) are more likely to be functionally similar (i.e., involved in a same biological process) than proteins that are further apart in the network [20]. Every cluster defined in this way would topologically be somewhere between a "star-like" network structure centered at the chosen SPR (as illustrated in Additional File 4 Figure S2 that presents the EDNRB cluster obtained by this "direct neighborhood" approach) and a fully connected network. That is, network clusters obtained with the direct neighborhood approach would have the diameter of either 1 or 2, where the diameter is defined as the maximum of shortest path lengths between all node pairs in the network. However, we tested *all *biological pathways from KEGG [21] mapped onto the human PPI network to compare their diameters with the diameters of the direct neighborhood-based clusters. Due to the incompleteness of the PPI network data, about 30% of KEGG pathways were consisting mainly of isolated edges after they were mapped onto the human PPI network; hence, we removed these pathways from our analysis. For the remaining 70% of the mapped pathways, we found that 93% of them have diameters larger than 2. The distribution of diameters of these pathways was bell-shaped, with the average of 6.24 and the standard deviation of 2.6. Therefore, since pathways correspond to parts of PPI networks that are of relatively large diameters, the simple direct neighborhood approach is not appropriate for finding new pathway members, since it can only produce subgraphs of small diameters. Hence, other approaches should be sought. In contrast, our GDV-based method generates EDNRB network of diameter 9. Also, since GDVs are based on all up to 5-node graphlets hence covering parts of the PPI network of diameter < 9 around an SPR of interest, the GDV-based approach is more appropriate for finding new pathway members than the direct neighborhoods.

With our GDV-based approach, in addition to the proteins in the cluster formed for a given SPR, we include into the SPR's "network" (defined above) the direct neighbors of proteins in the cluster to make the network connected; note that by doing so, we are indirectly taking into account the fact that direct neighbors are more likely to be involved in a same biological process than proteins that are further apart in the network [20]. For comparison, we perform the equivalent procedure for the direct neighborhood approach and evaluate whether this increases the diameters of such "expanded" direct neighborhood-based subnetworks. However, even if we include direct neighbors of the SPR's direct neighbors into the cluster, the diameter of the resulting networks would be between 1 and 4. Even if the resulting subnetworks would have the diameter of 4, that diameter is almost one standard deviation below the average diameter of known biological pathways (which is 6.24, as stated above). In contrast, our GDV-based approach produces PPI subnetworks with diameters between 5 and 9, with the average of 6.33 and the standard deviation of 1.5, thus mimicking well the diameters of known biological pathways.

### Validation of novel melanogenesis regulatory genes identified by topological clustering analysis

Topological clustering identified both SPRs and potentially novel pigment regulators that are components of the EDNRB network. As EDNRB and GNA11 siRNA impact pigment formation in our screen and regulate melanogenesis by well-characterized mechanisms, we next sought to experimentally validate novel EDNRB pathway components identified by our method. EDNRB, a g-protein coupled receptor mutated in Waardenburg-Shah syndrome [22], binds to EDN3 [23] and activates an intracellular signaling cascade via GNA11 [24]. Activation of EDNRB leads to PKC activation, and subsequent ERK phosphorylation. Phosphorylated ERK can either activate MITF by protein phosphorylation, or up-regulate MITF transcription through CREB phosphorylation [25,26]. MITF directly activates the expression of many melanogenesis regulators, such as tyrosinase, the rate-limiting enzyme in melanin synthesis [27]. In addition to regulating melanogenesis, EDNRB plays a critical role in neural crest development [28]. As some proteins that interact with EDNRB may impact neural crest development as opposed to melanogenesis, we focused on validating proteins in the EDNRB network most likely to directly impact melanogenesis. For this analysis, we select half of the SPRs most likely to directly regulate melanogenesis (Z-score < -1) from Figure 3: two SPRs in the cluster (EDNRB, CACNA1B), and four SPRs adjacent to the cluster (PTK2B, AGTR1, PLCD1, OPRM1). We then validate known regulators of pigmentation in our experimental system (PAX2, EDNRB, GNA11, EDN3), and examine other proteins in the EDNRB network not identified in our FG screen (FHOD1, DPYSL2, TAOK2, FHL1, RING1, CXCR4).

**Figure 4:**
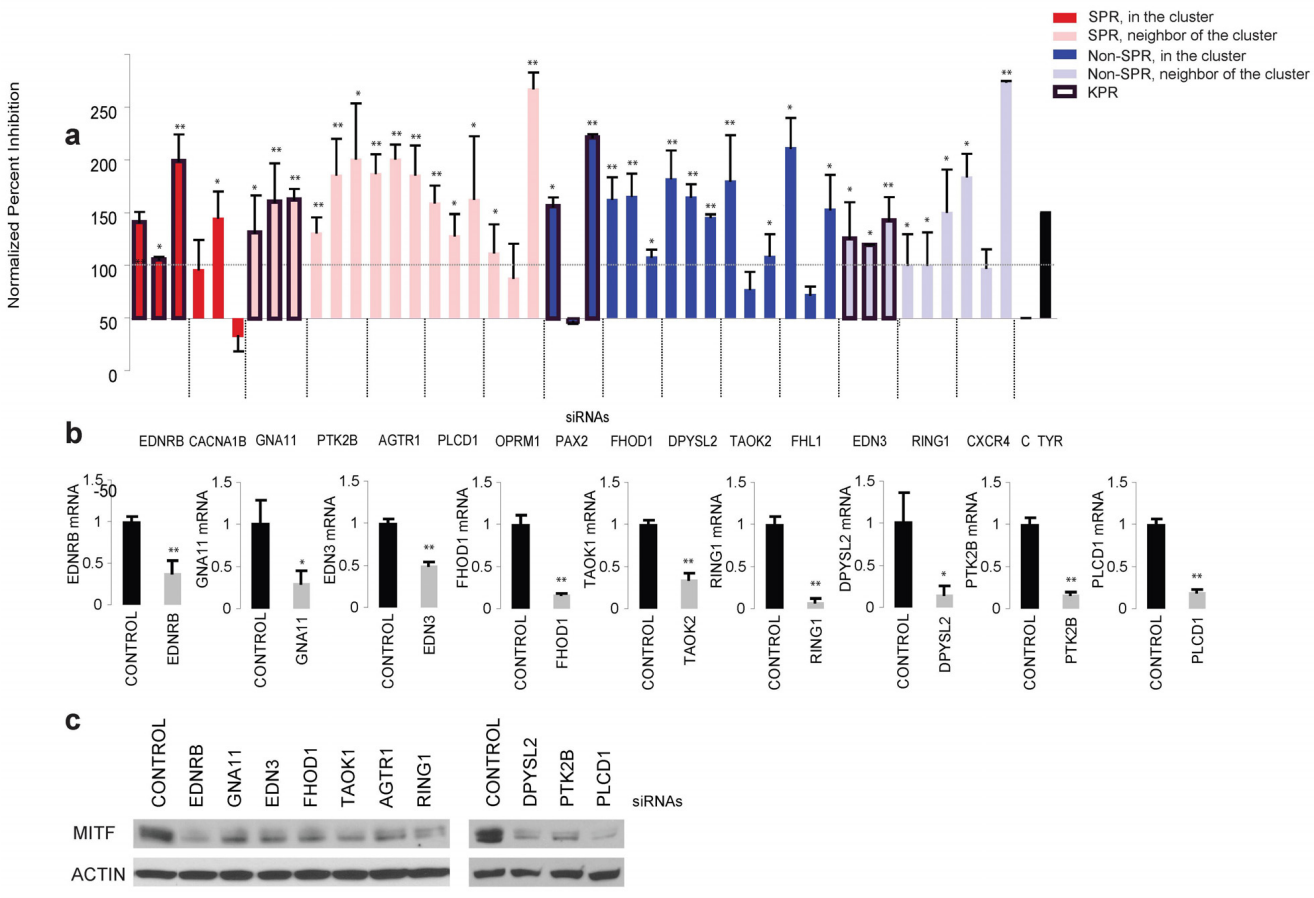
**Identification of novel EDNRB network components that impact melanogenesis**. (**a**) Genes from EDNRB network are examined for their impact on pigment production in MNT-1 cells. The impact of each siRNA on pigment production is calculated as described in [14] relative to control (c) and tyrosinase (TYR) siRNA treated cells using a normalized percent inhibition calculation. (**b**) Ten genes are selected from panel (a) that have > 50% NPI, and are not the result of siRNA off-target effects (2/3 of oligos have > 50% inhibition). The efficacy of protein knockdown for 10 pooled siRNAs directed towards these genes is measured by quantitative RT-PCR at both 24 and 48 hours after transfection. The timepoint where maximum inhibition of expression is observed is reported. AGTR1 mRNA level after siRNA treatment is below the detection limit and is not reported. Control siRNAs are depicted with a black bar, and target siRNAs with a white bar. *, *p *< 0.05. **, *p *< 0.01. (**c**) Both EDNRB and selected novel EDNRB network components impact the MITF protein levels in MNT-1 cells. MNT-1 cells are transfected with the indicated siRNAs for 96 hours followed by immunoblotting with MITF and ERK antibody as shown.

To experimentally validate our computational observations, we transfect MNT-1 cells with three siRNAs directed towards each gene or control siRNAs. The ability of each target siRNA to inhibit pigment production is compared to tyrosinase siRNA to control for the efficacy of siRNA transfection and the background absorbance of MNT-1 cells [13]. In this analysis, we utilize different siRNA sequences than those used in the initial FG screen (Additional File 3 Table S2) to further exclude any phenotypes from the initial screen that were the result of RNAi off-target effects. We also utilize a more robust cutoff to identify pigment regulators (~50% normalized pigment production) to identify only those genes with significant impacts on pigment production. SiRNAs targeting KPRs (EDNRB, GNA11, PAX2, EDN3) have impacts on pigment production, consistent with published studies. A majority of analyzed SPRs (86%) from EDNRB network (PTK2B, AGTR1, PLCD1, OPRM1) have significant impacts on pigment production observed with more than one oligonucleotide sequence, indicating that the phenotypes are not secondary to RNAi off-target effects (Figure 4a). Other proteins from EDNRB cluster (FHOD1, DPYSL2, TAOK2, FHL1) have a high retest rate as well (100%) (Figure 4a). The one gene that did not impact pigmentation in this analysis (CACNA1B) is a regulator of postganglionic synaptic transmission [29], an EDNRB neurologic phenotype independent of melanogenesis [30]. These results demonstrate that our EDNRB network contains newly defined regulators of pigment production (PTK2B, AGTR1, PLCD1, OPRM1, FHOD1, DPYSL2, TAOK2, FHL1, RING1, CXCR4).

### Impact of novel EDNRB pathway components on downstream signaling

**Figure 5:**
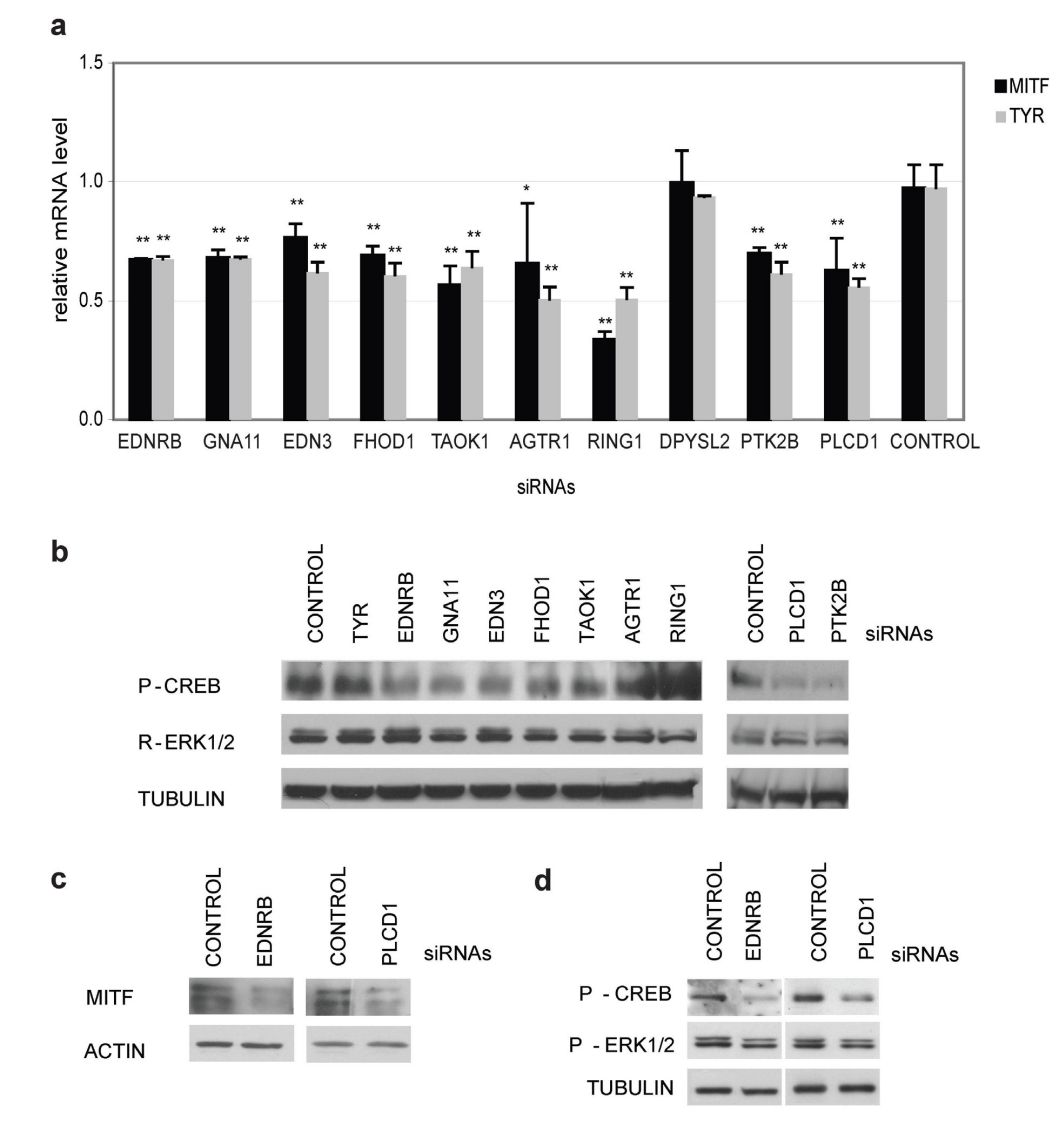
**Computationally derived EDNRB network components impact EDNRB signaling through CREB phosphorylation**. (**a**) Novel EDNRB network components impact both tyrosinase and MITF expression. MNT-1 melanoma cells are transfected with pooled siRNAs and relative MITF (black bar) and Tyrosinase (gray bar) expression is measured by quantitative RT-PCR 96 hours after transfection. *, *p *< 0.05. **, *p *< 0.01. (**b**) Novel components of the EDNRB network impact CREB phosphorylation. Lysates from MNT-1 cells treated with the indicated pooled siRNAs are subjected to immunoblotting with the indicated antibodies. (**c**) PLCD1 impacts MITF protein levels in melanocytes. Lysates from PLCD1 siRNA transfected melanocytes are subjected to immunoblotting with the indicated antibodies. (**d**) PLCD1 impacts CREB phophorylation in normal human melanocytes. Lysates from melanocytes transfected with the indicated siRNAs followed by EDNRB stimulation (treatment with 10 mM Endothelin-1 for 5 minutes) are subjected to immunoblotting with the indicated antibodies.

Our RNAi validation studies (Figure 4a) identified genes in the EDNRB network that impact pigment production. Based on functional annotation (Table 1), we next select 10 pigment regulators from Figure 4a to verify that these genes impact pigment production by impacting EDNRB signaling. Quantitative RT-PCR was utilized to determine the impact of each siRNA on its cognate target. RT-PCR validation studies were performed at two different timepoints to account for the potential differences in half-life of an individual mRNA. Our validation studies revealed that each siRNA inhibited the expression of the target gene at either 24 or 48 hours after siRNA transfection (Figure 4b). SiRNAs directed towards known EDNRB pathway components (EDNRB, EDN3, and GNA11) impact MITF protein levels, consistent with published studies [26] (Figure 4c). Similarly, novel EDNRB pathway components (FHOD1, TAOK1, AGTR1, RING1, DPYSL2, PLCD1) impact MITF protein levels, suggesting that these genes may have similar impacts on pigment production. Activation of the EDNRB pathway leads to increased MITF expression through p-CREB or leads to activation of MITF protein through phosphorylation [26]. In the simplest model, genes that impact MITF phosphorylation will impact tyrosinase expression while genes that impact MITF expression will impact both MITF and tyrosinase expression. Therefore, we would predict that siRNAs that inhibit MITF phosphorylation would have impacts on tyrosinase expression but not MITF expression, while siRNAs that impact CREB phosphorylation would inhibit both tyrosinase and MITF expression. To further validate this assertion, we examine the impact of each siRNA on the mRNA expression of MITF and tyrosinase. EDNRB, EDN3, and GNA11 have modest impacts on both MITF and tyrosinase expression, as predicted. Most of the other siRNAs examined impact on both MITF and tyrosinase expression, with the exception of DPYSL2 (Figure 5a).

**Table 1:** Annotation of genes identified in EDNRB network.

Symbol	Comments	Motifs	Z-score
EDNRB	Endothelin receptor type B. Mutation causes Hirschsprung disease type 2 (HSCR2) [MIM:600155]	7tm_1	-1.1

GNA11	G-protein activates phospholipase C in endothelin pathway	Gα	-1.9

EDN3	Ligand for endothelin receptor type B	EDN	0.2

PLCD1	Phospholipase produces diacylglycerol and inositol 1,4,5-trisphosphate in endothelin pathway	PH-PLC, C2_2, EFh	-1.5

TAOK1	Kinase activates the p38 MAP kinase pathway in endothelin pathway	SbcC, STKc_TAO	-0.2

PTK2B	Kinase regulates ion channel and activation of the MAP kinase signaling pathway in endothelin pathway	PTKc_FAK, FERM_M. Focal_AT, B41, Pkinase_Tyr	-1.9

AGTR1	Receptor for angiotensin II; defect causes renal tubular dysgenesis (RTD) [MIM:267430]	7tm_1	-1.7

RING1	E3 ubiquitin-protein ligase for histone H2A	RING-finger	0.5

FHOD1	Required for the assembly of F-actin structures	FH, GBD	0.2

DPYSL2	Signaling by class 3 semaphorins and subsequent remodeling of the cytoskeleton	D-HYD	-0.5

Gene Ontology Database is used to assess function and identify conserved domains within the corresponding proteins. Z-scores are calculated based on the initial screen data [14].

To determine the role of individual targets in EDNRB signaling, we examined whether the 9 genes (EDNRB, GNA11, EDN3, FHOD1, TAOK1, AGTR1, RING1, PTK2B, and PLCD1) that impact MITF and tyrosinase mRNA expression also impact CREB phosphorylation. Previous studies have determined that MNT-1 melanoma cells contain a BRAF mutation resulting in constitutive ERK activity [31]. Therefore, we would predict that the majority of EDNRB pathway components that impact pigment production in MNT-1 cells would do so via impacts on CREB phosphorylation, as the ERK pathway leading to MITF phosphorylation is constitutively active. Treatment of MNT-1 cells with EDNRB, EDN3, and GNA11 siRNAs impacts CREB phosphorylation but not ERK phosphorylation (Figure 5b). Similarly, TAOK1, PTK2B, FHOD1 and PLCD1 siRNAs inhibit CREB phosphorylation (Figure 5b). Published studies support the notion that these four genes are components of the EDNRB signaling network upstream of CREB phosphorylation. Serine/threonine-protein kinase TAO1 (TAOK1) activates the p38 MAP kinase pathway through the specific activation of the upstream MKK3 kinase [32]. This signaling pathway activates p38 leading to CREB phosphorylation in melanocytes [33,34]. It also is known that Protein tyrosine kinase 2 beta (PTK2B) activation leads to ERK1/2 activation [35]. In turn, ERK1/2 phosphorylates p90^rsk^, which phosphorylates CREB [34,36]. Endothelins promote dendrite formation facilitating melanosome transport from melanocytes to keratinocytes [37,38], processes that require extensive actin cytoskeletal reorganization [39,40]. Formin homology 2 domain containing 1 (FHOD1), a protein with several potential PKC phosphorylation sites [41], directly binds to F-actin and plays a role in actin cytoskeleton organization [42] and cell elongation [43]. Additionally, FHOD1 activates ERK inducing changes in gene expression [44]. Our studies implicate FHOD1 as a potential player in endothelin mediated actin-cytoskeletal reorganization.

Upon endothelins binding to endothelin receptors of melanocytes, phospholipase C is activated [45] and initiates subsequent activation of protein kinase C and ERK pathways. Phospholipase C delta-1 (PLCD1) hydrolyzes phosphatidylinositol 4,5-bisphosphate (PIP2) to generate two second messengers: diacylglycerol (DG) and inositol 1,4,5-trisphosphate (IP3) [46]. DG mediates the activation of protein kinase C (PKC), and IP3 releases calcium from intracellular stores, which in turns activate ERK [47]. In melanocytes, PLC antagonist U73122 inhibits endothelin-1-induced intracellular calcium rise and ERK phosphorylation [48,49], providing direct evidence that phospholipase C activity impacts endothelin-induced signaling in primary melanocytes. Analysis of gene annotation data reveals that PLCD1 is a phospholipase C homologue that may function directly downstream of GNA11 (Table 1). As PLC activity impacts EDNRB signaling in melanocytes, we sought to examine whether PLCD1 was the PLC isoform that impacts EDNRB signaling in normal melanocytes. Depletion of PLCD1 in normal melanocytes impacts MITF protein levels (Figure 5c). Additionally, we demonstrate that PLCD1 siRNA inhibits CREB phosphorylation in the context of EDNRB receptor stimulation, consistent with the hypothesis that PLCD1 is upstream of the CREB phosphorylation event. These results demonstrate that some components in our EDNRB network participate in EDNRB signaling in normal and cancer cells (Figure 5d).

In this study, we apply a PPI network topology-based approach that utilizes GDVs to identify novel components of melanogenesis regulatory pathways. Related approaches that detect biological function from PPI network topology rely on simpler network properties such as direct neighbors of nodes and shortest path distances [20,50]. Since none of the 6 SPRs in EDNRB cluster are adjacent in the PPI network, but are at distance three or four from each other, they could not have been topologically clustered together by analyzing only their neighbors or distances in the PPI network. Additionally, as mentioned above, examining only direct neighbors of an SPR is not appropriate for constructing its underlying melanogenesis-related pathway.

Although GDV-based computational method has already been used to demonstrate that topologically similar proteins share common biological properties such as protein function or involvement in cancer [17,18], in this paper, we provide a novel application of the method, namely identifying novel components of biological pathways known to regulate melanogenesis. We use this graph theoretic method to identify proteins with similar topological signatures to known pigment regulators and to construct the corresponding melanogenesis-related subnetworks. Most importantly, we experimentally validate that the novel components of these melanogenesis-related subnetworks are components of the EDNRB pathway in both pigmented melanoma cells and normal melanocytes.

## Conclusions

In this study, we utilize a PPI network topology-based computational approach to identify a set of novel EDNRB pathway components. Using RNAi-based approaches, we validate that some of the proteins in EDNRB network impact melanogenesis, demonstrating that topological clustering approaches can uncover additional components of known regulatory networks. SPRs that cluster with EDNRB have similar impacts on MITF protein levels, on MITF and tyrosinase expression, and on CREB phosphorylation, indicating that they are likely components of the same biological pathway. Validation of PLCD1, a potent regulator of MITF in MNT-1 cells, reveals that novel EDNRB pathway component identified in MNT-1 cells impact EDNRB signaling in normal melanocytes. Our study demonstrates that integration of PPI network topology-based algorithms with functional genomics datasets is a fruitful method to enhance our systems-level understanding of biological phenotypes. In the future this method may be used to place targets identified in RNAi screens within the context of known biological pathways that regulate the system under study, deriving additional relevance from systems-level RNAi datasets.

## Methods

### Cell culture

MNT-1 melanoma cells were a gift of M. Marks (University of Pennylvania) and were cultured in DMEM (Invitrogen) with 15% fetal bovine serum (Hyclone), 10% AIM-V medium (Invitrogen), 1xMEM (Invitrogen) and 1 × antibiotic/antimycotic (Invitrogen). Darkly pigmented human neonatal epidermal melanocytes (Cascade Biologics) were cultured in Medium 254 with the melanocyte specific HMGS supplement (Cascade Biologics) and 0.1 mM 3-Isobutyl-1-methylxanthine (IBMX).

### Protein clustering via topological similarity measure

In our study, we focus on proteins with more than three interacting partners in the PPI network, since poorly connected proteins are more likely to reside in incomplete parts of PPI networks [17,18]. However, to account for the entire topology of the PPI network, when GDVs are computed, all proteins and interactions in the PPI network are taken into account. There are 5,423 proteins with degrees higher than three in the network, out of which 1,244 are SPRs, and 41 are KPRs contained in the ESPCR database [8]; 10 SPRs are KPRs at the same time. The code for computing GDVs [17] is available in GraphCrunch [51], an open source software tool for large network modeling and analyses.

We perform the GDV-based clustering as follows: for each of the 1,244 SPRs with degrees greater than 3 in the PPI network, we identify a cluster containing that protein and all other proteins in the network having degrees higher than 3 that are topologically similar to it, i.e., that have GDV similarities with the protein of interest above a threshold; thresholds between 90% and 95% have been used previously [17]. We find the highest threshold at which a node is clustered with at least one more node by performing the following: (1) we initially set the signature similarity threshold to 96%; (2) if the SPR for which the cluster is formed is signature-similar with at least one more node at the given threshold, we create the cluster containing these nodes and the SPR; (3) if a protein of interest is not signature-similar with any other protein at a given threshold, we decrease the threshold and repeat steps 2 and 3; (4) we stop the clustering process for a given SPR when its cluster has been formed; we decrease the threshold down to 90% searching for non-empty clusters, but not below 90%; if a protein is not signature-similar with any other protein at the lowest threshold of 90%, we do not form a cluster for that protein. We try to form clusters for all 1,244 SPRs. However, 263 of them are not 90% signature similar to any other nodes in the PPI network. Additional 237 of them cluster only with one other node. Thus, we analyze clusters of size 3 or more formed for 743 SPRs. The minimum threshold of 90% for signature similarity was chosen because it has been empirically shown that for higher thresholds, only a few small clusters are obtained, indicating too high stringency in signature similarities, whereas for lower thresholds, clusters are very large, indicating a loss of signature similarity [17].

In each cluster formed in this fashion, we measure the enrichment of SPRs. We define the "hit-rate" of a cluster as the percentage of proteins in the cluster that are SPRs, excluding the protein of interest. We also compute the statistical significance of observing a given hit-rate in the cluster, measuring the probability that the cluster is enriched by a given number of SPR proteins purely by chance. This probability (*p*-value) is computed as follows. The total number of proteins in the PPI network with degrees higher than 3 is *N *= 5,422 (excluding the SPR for which the cluster was formed) and there are *S *= 1,243 SPRs with degrees higher than 3 (excluding the SPR for which the cluster was formed). We use the following notation: the size of a cluster of interest is *C*, excluding the protein for which the cluster was formed; the number of proteins in the cluster that are SPRs is *k*, excluding the protein for which the cluster was formed. The hit-rate of the cluster is *k/|C|*, and the *p*-value for the cluster, i.e., the probability of observing the same or higher hit-rate purely by chance is:

Depending on a method and its application, sensible cut-offs for *p*-values were reported to range from 10^-2 ^to 10^-8 ^[52]. We find that 26 out of 743 clusters formed for SPRs are enriched with SPRs with p-values of SPR enrichment below 0.05. Since we perform statistical testing on 743 clusters, we believe that the reported p-values do not need to be corrected for multiple testing.

Given that 26 of our clusters are statistically significantly SPR-enriched at p-value threshold of 0.05, we compute the probability of observing 26 statistically significantly SPR-enriched clusters by chance (i.e., statistical significance). For each of the 743 SPRs, we create "random" clusters of the same size as the corresponding GDV-based clusters. Then, we measure statistical significance of SPR enrichment in these random clusters. Since the clusters are random, we repeat the randomization procedure 1,000 times. We find that only in 9 out of the 1,000 runs, 26 or more clusters are statistically significantly enriched with SPRs, leading to empirical (frequency) probability of 0.009 for observing 26 or more statistically significantly SPR-enriched clusters by chance. Furthermore, since the distribution of the number of statistically significantly SPR-enriched random cluster over the 1,000 runs is bell-shaped, we use the formula for computing Z-score to standardize our observation in the data of having 26 statistically significantly SPR-enriched clusters. Then, we use the normal distribution table to compute the p-value corresponding to the resulting Z-score. If we denote by x = 26 the raw score to be standardized, by μ= 15.45 the mean of the (random) population, i.e., the average over the 1,000 runs of the number of random clusters that are statistically significantly SPR-enriched, and by σ = 4.03 the standard deviation over the 1,000 runs, we get Z-score of 2.62. Using the normal distribution table, we find that this Z-score corresponds to the p-value of 0.0044; that is, in this way, we find that the probability of observing 26 or more statistically significantly SPR-enriched clusters at random is lower than 0.0044.

### SiRNA transfection

For western blot analysis, 6 × 10^4 ^MNT-1 cells were transfected with 75 nM pooled siRNAs using a Dharmafect-2 transfection reagent as previously described [13]. Similarly, 6 × 10^4 ^melanocytes were transfected with 12.5 nM pooled siRNAs (two) toward each gene using a HiPerFect transfection reagent as previously described [13]. For pigment measurement, 1 × 10^4 ^MNT-1 cells were transfected with 8 nM pooled siRNAs (two siRNAs) using a Dharmafect-2 transfection reagent as described. We performed each transfection in triplicate. For RT-qPCR, we used 4 × 10^3 ^MNT-1 cells in the same 96-well format. We obtained pooled siRNAs for tyrosinase from Dharmacon, and all the other siRNAs from Ambion. A list of all siRNA sequences are contained in Additional File 5 Table S3.

### Pigment measurement and data normalization

For pigment measurement, we transfected MNT-1 cells with the corresponding siRNA in 96 well plates and incubated them for 120 hours at 37°C/5% CO2. Subsequently, we removed 100 ul of the medium from each well and added 15 ul of CellTiter-Glo Reagent (CTG) (Promega) to each well according to the manufacturer's protocol. We measured luminescence and absorbance at 405 nm for each well of the 96 well plate using a DTX800 Multimode Detector (Beckman Coulter). We normalized absorbance values with raw luminescence values that are proportional to cell number to determine relative pigment per cell. We calculated a normalized percent inhibition from these values to account for the background absorbance of MNT-1 cells and variability in transfection. Normalized percent inhibition (NPI) = [(Pigment Index _non-targeting control siRNA _-Pigment Index _sample_)/(Pigment Index _non-targeting control _-Pigment Index _tyrosinase _siRNA)] × 100%. We use a student's t-test to calculate the *p*-values of each sample v.s. non-targeting control.

### Immunoblotting

For our immunoblotting experiments, MNT-1 cells 96 hrs post-transfection and melanocytes 144hrs post-transfection were lysed in RIPA buffer supplied with proteases inhibitors cocktail, PMSF and sodium orthovanadate (Santa Cruz Biotechnology). For experiments in melanocytes looking at downstream EDNRB signaling, we treated melanocytes with 10 μM Endothelin-1 for 5mins. After boiling with sample buffer, we loaded the samples to 4-20% SDS-PAGE and subjected them to western blotting with anti-MITF (C5, Santa Cruz Biotechnology), anti-phospho-ERK1/2 (Cell Signaling Technologics), anti-phospho-CREB (Cell Signaling Technologics), anti-alpha/beta-tubulin (Cell Signaling Technologics) and anti-beta-actin (Cell Signaling Technologics) antibodies.

### Quantitative RT-PCR

96hrs after siRNA transfection, we prepared cDNA from MNT-1 cells using a Cells-to-Ct kit (Ambion) according to the manufacterer's protocol. For measurement of siRNA knockdown efficiency, we prepared cDNA from MNT-1 cells 48 hrs after transfection for most of the genes except RING1, FHOD1, TAOK1 at 24 hrs to capture the impact in different mRNA kinetics. We added an aliquot of each cDNA reaction to each Taqman master mix reaction along with the appropriate primer and probe set purchased from Applied Biosystems per the manufacturer's protocol (Applied Biosystems). Primers and probes sequences are listed in Additional File 6 Table S4. We utilized a 7900HT Fast Real-Time PCR System (Applied Biosystems) to determine Ct values. We normalized values by human beta-actin Ct values and analyzed our data using the relative quantification mathematical model (Pfaffl).

## Abbreviations

RNAi: RNA-mediated interference; siRNA: small interfering RNA; RT-PCR: reverse-transcription polymerase chain reaction; PPI: protein-protein interaction; FG: functional genomics; EDNRB: Endothelin receptor type B; MITF: microphthalmia-associated transcription factor; SPR: screen pigment regulator; KPR: known pigment regulator; GDV: graphlet degree vector; CREB: cAMP response element binding; ERK: extracellular signal-regulated kinase.

## Authors' contributions

TM and VM performed the computational data analysis. HH and JA performed biological experiments. AKG and NP conceived of the study. All the authors participated in its design and coordination. AKG, NP and HH drafted the manuscript. All authors read and approved the final manuscript.

## Supplementary Material

Additional file 1 **Figure S1: (a) All 30 graphlets on 2 to 5 nodes; they contain 73 topologically unique node types, called "automorphism orbits"**. In a particular graphlet, nodes belonging to the same orbit are of the same shade [52]. (**b**) An illustration of the "Graphlet Degree Vector" (GDV), or a "signature" of node *v*; coordinates of a GDV count how many times a node is touched by a particular automorphism orbit, such as an edge (the leftmost panel), a triangle (the middle panel), or a square (the rightmost panel). Hence, the degree is generalized to a GDV [17]. The GDV of node v is presented in the table for orbits 0 to 72: v is touched by 5 edges (orbit 0), end-nodes of 2 graphlets G_1 _(orbit 1), etc. Click here for file

Additional file 2 **Table S1: siRNAs from our genome wide siRNA screen with Z-score < -1 or > 1**. Click here for file

Additional file 3 **Table S2: Proteins that form the cluster**. Click here for file

Additional file 4 **Figure S2: EDNRB cluster obtained by considering its direct neighbors in the PPI network**. Unlike the EDNRB network obtained with our GDV-based approach, this network is clearly a star-like network structure centered around EDNRB. This structure is unlikely to represent a biological pathway, since its diameter, defined as the maximum of shortest path distances between all node pairs in the network, is 2, whereas we find that about 93% of real human biological pathways from KEGG [21], when mapped onto the human PPI network, have diameters larger than 2, with the average of 6.24 and the standard deviation of 2.6. Click here for file

Additional file 5 **Table S3: siRNA sequences used in this study**. Click here for file

Additional file 6 **Table S4: qPCR probe sequences used in this study**. Click here for file

## References

[B1] Lehner B Lee I Network-guided genetic screening: building, testing and using gene networks to predict gene function Brief Funct Genomic Proteomic 2008 7 217 227 10.1093/bfgp/eln020 18445637

[B2] Lamitina T Functional genomic approaches in C. elegans Methods Mol Biol 2006 351 127 138 16988431 10.1385/1-59745-151-7:127

[B3] Gunsalus KC Ge H Schetter AJ Goldberg DS Han JD Hao T Berriz GF Bertin N Huang J Chuang LS Predictive models of molecular machines involved in Caenorhabditis elegans early embryogenesis Nature 2005 436 861 865 10.1038/nature03876 16094371

[B4] Lee K Chuang HY Beyer A Sung MK Huh WK Lee B Ideker T Protein networks markedly improve prediction of subcellular localization in multiple eukaryotic species Nucleic Acids Res 2008 36 e136 10.1093/nar/gkn619 18836191 PMC2582614

[B5] Kaplow IM Singh R Friedman A Bakal C Perrimon N Berger B RNAiCut: automated detection of significant genes from functional genomic screens Nat Methods 2009 6 476 477 10.1038/nmeth0709-476 19564846

[B6] Echeverri CJ Beachy PA Baum B Boutros M Buchholz F Chanda SK Downward J Ellenberg J Fraser AG Hacohen N Minimizing the risk of reporting false positives in large-scale RNAi screens Nat Methods 2006 3 777 779 10.1038/nmeth1006-777 16990807

[B7] Konig R Zhou Y Elleder D Diamond TL Bonamy GM Irelan JT Chiang CY Tu BP De Jesus PD Lilley CE Global analysis of host-pathogen interactions that regulate early-stage HIV-1 replication Cell 2008 135 49 60 10.1016/j.cell.2008.07.032 18854154 PMC2628946

[B8] Bennett DC Lamoreux ML The color loci of mice--a genetic century Pigment Cell Res 2003 16 333 344 10.1034/j.1600-0749.2003.00067.x 12859616

[B9] Slominski A Tobin DJ Shibahara S Wortsman J Melanin pigmentation in mammalian skin and its hormonal regulation Physiol Rev 2004 84 1155 1228 10.1152/physrev.00044.2003 15383650

[B10] Levy C Khaled M Fisher DE MITF: master regulator of melanocyte development and melanoma oncogene Trends Mol Med 2006 12 406 414 10.1016/j.molmed.2006.07.008 16899407

[B11] Raposo G Marks MS Melanosomes--dark organelles enlighten endosomal membrane transport Nat Rev Mol Cell Biol 2007 8 786 797 10.1038/nrm2258 17878918 PMC2786984

[B12] Sulem P Gudbjartsson DF Stacey SN Helgason A Rafnar T Magnusson KP Manolescu A Karason A Palsson A Thorleifsson G Genetic determinants of hair, eye and skin pigmentation in Europeans Nat Genet 2007 39 1443 1452 10.1038/ng.2007.13 17952075

[B13] Ganesan AK Ho H Bodemann B Petersen S Aruri J Koshy S Richardson Z Le LQ Krasieva T Roth MG Genome-wide siRNA-based functional genomics of pigmentation identifies novel genes and pathways that impact melanogenesis in human cells PLoS Genet 2008 4 e1000298 10.1371/journal.pgen.1000298 19057677 PMC2585813

[B14] Peri S Navarro JD Kristiansen TZ Amanchy R Surendranath V Muthusamy B Gandhi TK Chandrika KN Deshpande N Suresh S Human protein reference database as a discovery resource for proteomics Nucleic Acids Res 2004 32 D497 501 10.1093/nar/gkh070 14681466 PMC308804

[B15] Stark C Breitkreutz BJ Reguly T Boucher L Breitkreutz A Tyers M BioGRID: a general repository for interaction datasets Nucleic Acids Res 2006 34 D535 539 10.1093/nar/gkj109 16381927 PMC1347471

[B16] Radivojac P Peng K Clark WT Peters BJ Mohan A Boyle SM Mooney SD An integrated approach to inferring gene-disease associations in humans Proteins 2008 72 1030 1037 10.1002/prot.21989 18300252 PMC2824611

[B17] Milenković T Pržulj N Uncovering Biological Network Function via Graphlet Degree Signatures Cancer Inform 2008 6 257 273 19259413 PMC2623288

[B18] Milenković TVM Ganesan AK Pržulj N Systems-level cancer gene identification from protein interaction network topology applied to melanogenesis-related functional genomics data J R Soc Interface 2010 10.1098/rsif.2009.0192 PMC2842789 19625303

[B19] Motulsky H Intuitive Biostatistics 1995 Oxford University Press, New York

[B20] Sharan R Ulitsky I Shamir R Network-based prediction of protein function Mol Syst Biol 2007 3 88 10.1038/msb4100129 17353930 PMC1847944

[B21] Kanehisa M Goto S KEGG: kyoto encyclopedia of genes and genomes Nucleic Acids Res 2000 28 27 30 10.1093/nar/28.1.27 10592173 PMC102409

[B22] Shanske A Ferreira JC Leonard JC Fuller P Marion RW Hirschsprung disease in an infant with a contiguous gene syndrome of chromosome 13 Am J Med Genet 2001 102 231 236 10.1002/ajmg.1451 11484199

[B23] Hofstra RM Osinga J Tan-Sindhunata G Wu Y Kamsteeg EJ Stulp RP van Ravenswaaij-Arts C Majoor-Krakauer D Angrist M Chakravarti A A homozygous mutation in the endothelin-3 gene associated with a combined Waardenburg type 2 and Hirschsprung phenotype (Shah-Waardenburg syndrome) Nat Genet 1996 12 445 447 10.1038/ng0496-445 8630503

[B24] Van Raamsdonk CD Fitch KR Fuchs H de Angelis MH Barsh GS Effects of G-protein mutations on skin color Nat Genet 2004 36 961 968 10.1038/ng1412 15322542 PMC7341985

[B25] McGill GG Horstmann M Widlund HR Du J Motyckova G Nishimura EK Lin YL Ramaswamy S Avery W Ding HF Bcl2 regulation by the melanocyte master regulator Mitf modulates lineage survival and melanoma cell viability Cell 2002 109 707 718 10.1016/S0092-8674(02)00762-6 12086670

[B26] Sato-Jin K Nishimura EK Akasaka E Huber W Nakano H Miller A Du J Wu M Hanada K Sawamura D Epistatic connections between microphthalmia-associated transcription factor and endothelin signaling in Waardenburg syndrome and other pigmentary disorders Faseb J 2008 22 1155 1168 10.1096/fj.07-9080com 18039926

[B27] Murisier F Beermann F Genetics of pigment cells: lessons from the tyrosinase gene family Histol Histopathol 2006 21 567 578 16493586 10.14670/HH-21.567

[B28] Hou L Pavan WJ Shin MK Arnheiter H Cell-autonomous and cell non-autonomous signaling through endothelin receptor B during melanocyte development Development 2004 131 3239 3247 10.1242/dev.01193 15201217

[B29] Motagally MA Lukewich MK Chisholm SP Neshat S Lomax AE Tumour necrosis factor alpha activates nuclear factor kappaB signalling to reduce N-type voltage-gated Ca2+ current in postganglionic sympathetic neurons J Physiol 2009 587 2623 2634 10.1113/jphysiol.2009.172312 19403618 PMC2714026

[B30] Barlow A de Graaff E Pachnis V Enteric nervous system progenitors are coordinately controlled by the G protein-coupled receptor EDNRB and the receptor tyrosine kinase RET Neuron 2003 40 905 916 10.1016/S0896-6273(03)00730-X 14659090

[B31] Hoek K Rimm DL Williams KR Zhao H Ariyan S Lin A Kluger HM Berger AJ Cheng E Trombetta ES Expression profiling reveals novel pathways in the transformation of melanocytes to melanomas Cancer Res 2004 64 5270 5282 10.1158/0008-5472.CAN-04-0731 15289333

[B32] Yustein JT Xia L Kahlenburg JM Robinson D Templeton D Kung HJ Comparative studies of a new subfamily of human Ste20-like kinases: homodimerization, subcellular localization, and selective activation of MKK3 and p38 Oncogene 2003 22 6129 6141 10.1038/sj.onc.1206605 13679851

[B33] Saha B Singh SK Sarkar C Bera R Ratha J Tobin DJ Bhadra R Activation of the Mitf promoter by lipid-stimulated activation of p38-stress signalling to CREB Pigment Cell Res 2006 19 595 605 10.1111/j.1600-0749.2006.00348.x 17083486

[B34] Tada A Pereira E Beitner-Johnson D Kavanagh R Abdel-Malek ZA Mitogen-and ultraviolet-B-induced signaling pathways in normal human melanocytes J Invest Dermatol 2002 118 316 322 10.1046/j.0022-202x.2001.01694.x 11841550

[B35] Lev S Moreno H Martinez R Canoll P Peles E Musacchio JM Plowman GD Rudy B Schlessinger J Protein tyrosine kinase PYK2 involved in Ca(2+)-induced regulation of ion channel and MAP kinase functions Nature 1995 376 737 745 10.1038/376737a0 7544443

[B36] Bohm M Moellmann G Cheng E Alvarez-Franco M Wagner S Sassone-Corsi P Halaban R Identification of p90RSK as the probable CREB-Ser133 kinase in human melanocytes Cell Growth Differ 1995 6 291 302 7540859

[B37] Demunter A De Wolf-Peeters C Degreef H Stas M van den Oord JJ Expression of the endothelin-B receptor in pigment cell lesions of the skin. Evidence for its role as tumor progression marker in malignant melanoma Virchows Arch 2001 438 485 491 10.1007/s004280000362 11407477

[B38] Hara M Yaar M Gilchrest BA Endothelin-1 of keratinocyte origin is a mediator of melanocyte dendricity J Invest Dermatol 1995 105 744 748 10.1111/1523-1747.ep12325522 7490466

[B39] Busca R Bertolotto C Abbe P Englaro W Ishizaki T Narumiya S Boquet P Ortonne JP Ballotti R Inhibition of Rho is required for cAMP-induced melanoma cell differentiation Mol Biol Cell 1998 9 1367 1378 9614180 10.1091/mbc.9.6.1367 PMC25356

[B40] Hata K Mukaiyama T Tsujimura N Sato Y Kosaka Y Sakamoto K Hori K Differentiation-inducing activity of lupane triterpenes on a mouse melanoma cell line Cytotechnology 2006 52 151 158 10.1007/s10616-007-9069-0 19002873 PMC3449405

[B41] Westendorf JJ Mernaugh R Hiebert SW Identification and characterization of a protein containing formin homology (FH1/FH2) domains Gene 1999 232 173 182 10.1016/S0378-1119(99)00127-4 10352228

[B42] Takeya R Sumimoto H Fhos, a mammalian formin, directly binds to F-actin via a region N-terminal to the FH1 domain and forms a homotypic complex via the FH2 domain to promote actin fiber formation J Cell Sci 2003 116 4567 4575 10.1242/jcs.00769 14576350

[B43] Gasteier JE Schroeder S Muranyi W Madrid R Benichou S Fackler OT FHOD1 coordinates actin filament and microtubule alignment to mediate cell elongation Exp Cell Res 2005 306 192 202 10.1016/j.yexcr.2005.02.006 15878344

[B44] Boehm MB Milius TJ Zhou Y Westendorf JJ Koka S The mammalian formin FHOD1 interacts with the ERK MAP kinase pathway Biochem Biophys Res Commun 2005 335 1090 1094 10.1016/j.bbrc.2005.07.191 16112087

[B45] Tanaka H Moroi K Iwai J Takahashi H Ohnuma N Hori S Takimoto M Nishiyama M Masaki T Yanagisawa M Novel mutations of the endothelin B receptor gene in patients with Hirschsprung's disease and their characterization J Biol Chem 1998 273 11378 11383 10.1074/jbc.273.18.11378 9556633

[B46] Stallings JD Tall EG Pentyala S Rebecchi MJ Nuclear translocation of phospholipase C-delta1 is linked to the cell cycle and nuclear phosphatidylinositol 4,5-bisphosphate J Biol Chem 2005 280 22060 22069 10.1074/jbc.M413813200 15809301

[B47] Kobayashi A Hachiya A Ohuchi A Kitahara T Takema Y Inhibitory mechanism of an extract of Althaea officinalis L. on endothelin-1-induced melanocyte activation Biol Pharm Bull 2002 25 229 234 10.1248/bpb.25.229 11853172

[B48] Imokawa G Kobayasi T Miyagishi M Intracellular signaling mechanisms leading to synergistic effects of endothelin-1 and stem cell factor on proliferation of cultured human melanocytes. Cross-talk via trans-activation of the tyrosine kinase c-kit receptor J Biol Chem 2000 275 33321 33328 10.1074/jbc.M004346200 10921922

[B49] Kang HY Kang WH Lee C Endothelin-B receptor-mediated Ca2+ signaling in human melanocytes Pflugers Arch 1998 435 350 356 9426289 10.1007/pl00008082

[B50] Pržulj N Milenković T Jake Chen, Stefano Lonardi Computational Methods for Analyzing and Modeling Biological Networks, a chapter in "Biological Data Mining" 2009 Chapman & Hall/CRC

[B51] Milenković T Lai J Pržulj N GraphCrunch: a tool for large network analyses BMC Bioinformatics 2008 70 http://www.ics.uci.edu/~bio-nets/graphcrunch/10.1186/1471-2105-9-70 18230190 PMC2275247

[B52] King AD Pržulj N Jurisica I Protein complex prediction via cost-based clustering Bioinformatics 2004 20 3013 3020 10.1093/bioinformatics/bth351 15180928

