# Supplementary figures and images for: Protein interaction network topology uncovers melanogenesis regulatory network components within functional genomics datasets
Source: BMC Syst Biol. 2010 Jun 15;4:84. doi: 10.1186/1752-0509-4-84 (PMC2904735; doi:10.1186/1752-0509-4-84)

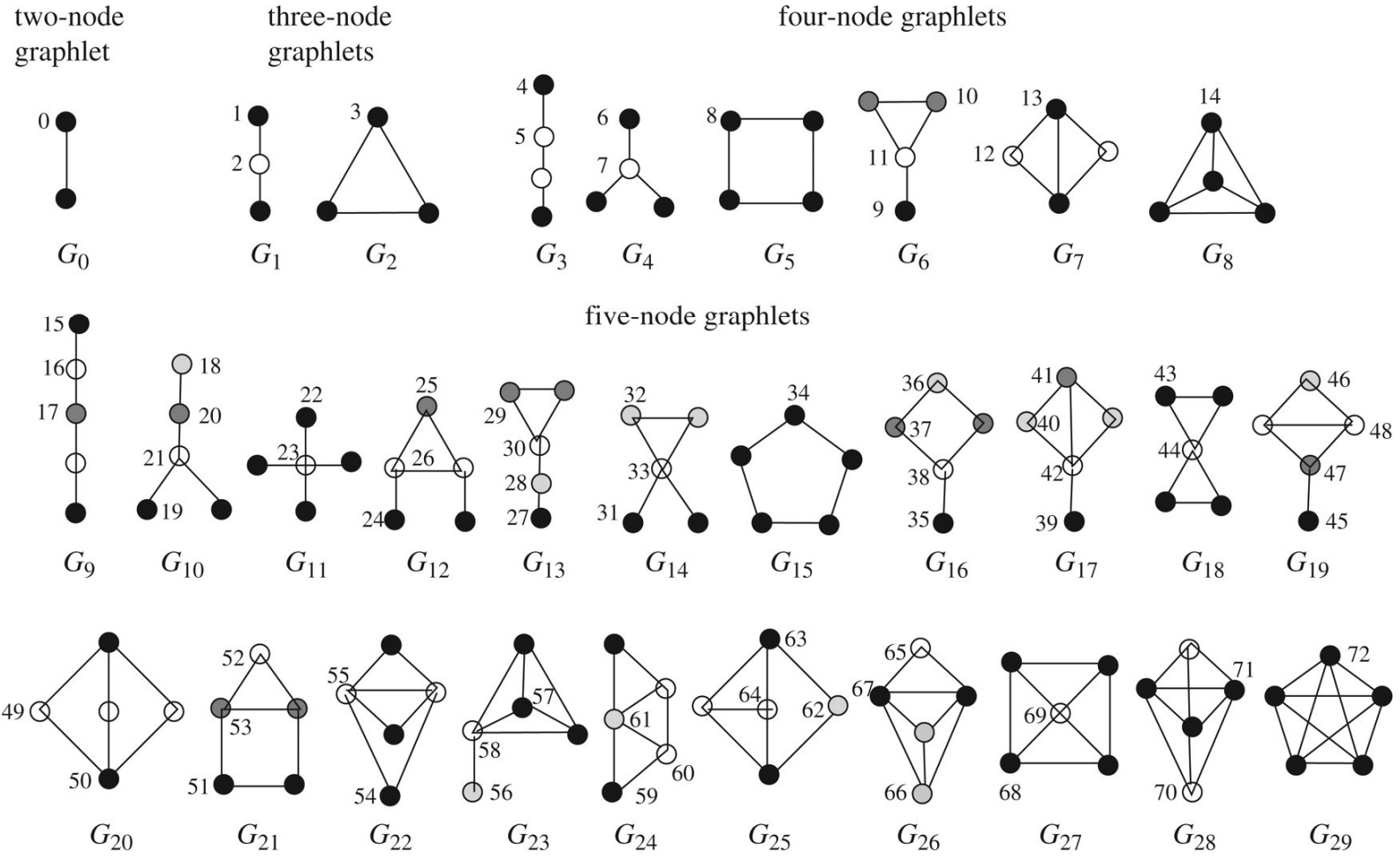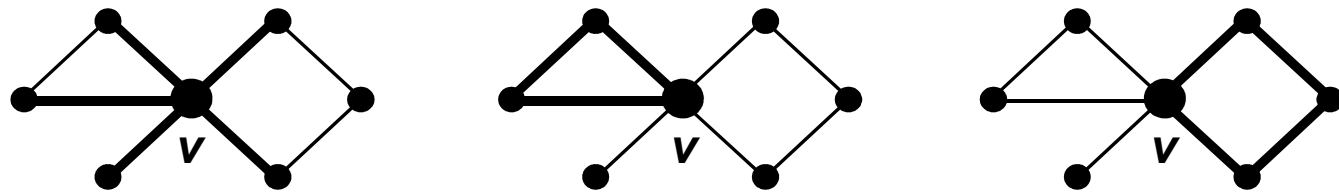

| Orbit       | 0 | 1 | 2 | 3 | 4 | 5 | 6 | 7 | 8 | 9 | 10 | 11 | 12...20 | 21 | 22 | 23 | 24...29 | 30 | 31 | 32 | 33 | 34...37 | 38 | 39...72 |
|-------------|---|---|---|---|---|---|---|---|---|---|----|----|---------|----|----|----|---------|----|----|----|----|---------|----|---------|
| GDV ( $v$ ) | 5 | 2 | 9 | 1 | 0 | 6 | 0 | 7 | 1 | 0 | 0  | 3  | 0       | 4  | 0  | 2  | 0       | 2  | 0  | 0  | 3  | 0       | 3  | 0       |

(b)

Supplement: Additional file 1 — Figure S1: (a) All 30 graphlets on 2 to 5 nodes; they contain 73 topologically unique node types, called "automorphism orbits". In a particular graphlet, nodes belonging to the same orbit are of the same shade [52]. (b) An illustration of the "Graphlet Degree Vector" (GDV), or a "signature" of node v; coordinates of a GDV count how many times a node is touched by a particular automorphism orbit, such as an edge (the leftmost panel), a triangle (the middle panel), or a square (the rightmost panel). Hence, the degree is generalized to a GDV [17]. The GDV of node v is presented in the table for orbits 0 to 72: v is touched by 5 edges (orbit 0), end-nodes of 2 graphlets G1 (orbit 1), etc. [file 1752-0509-4-84-S1.PDF]

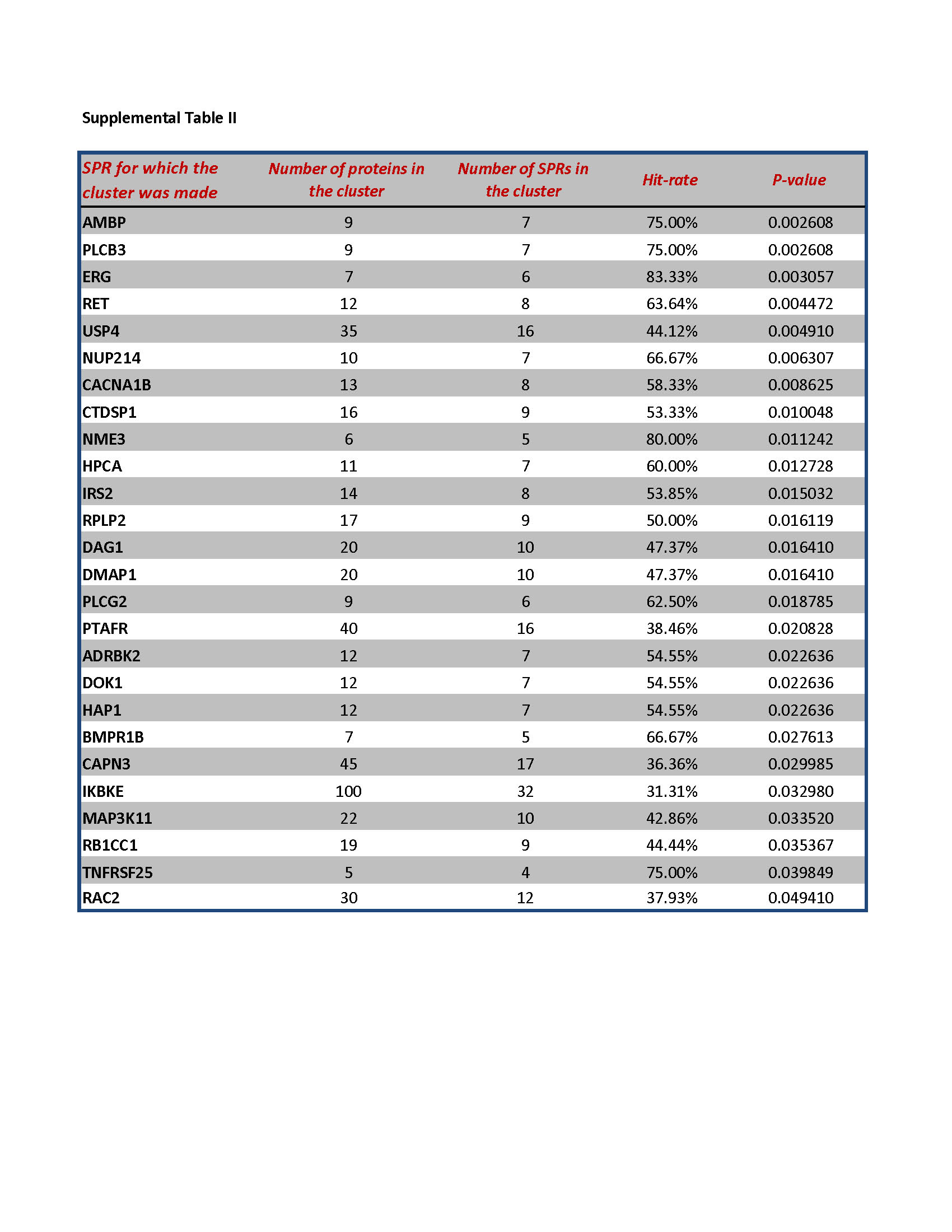

Supplement: Additional file 3 — Table S2: Proteins that form the cluster. [file 1752-0509-4-84-S3.JPEG]

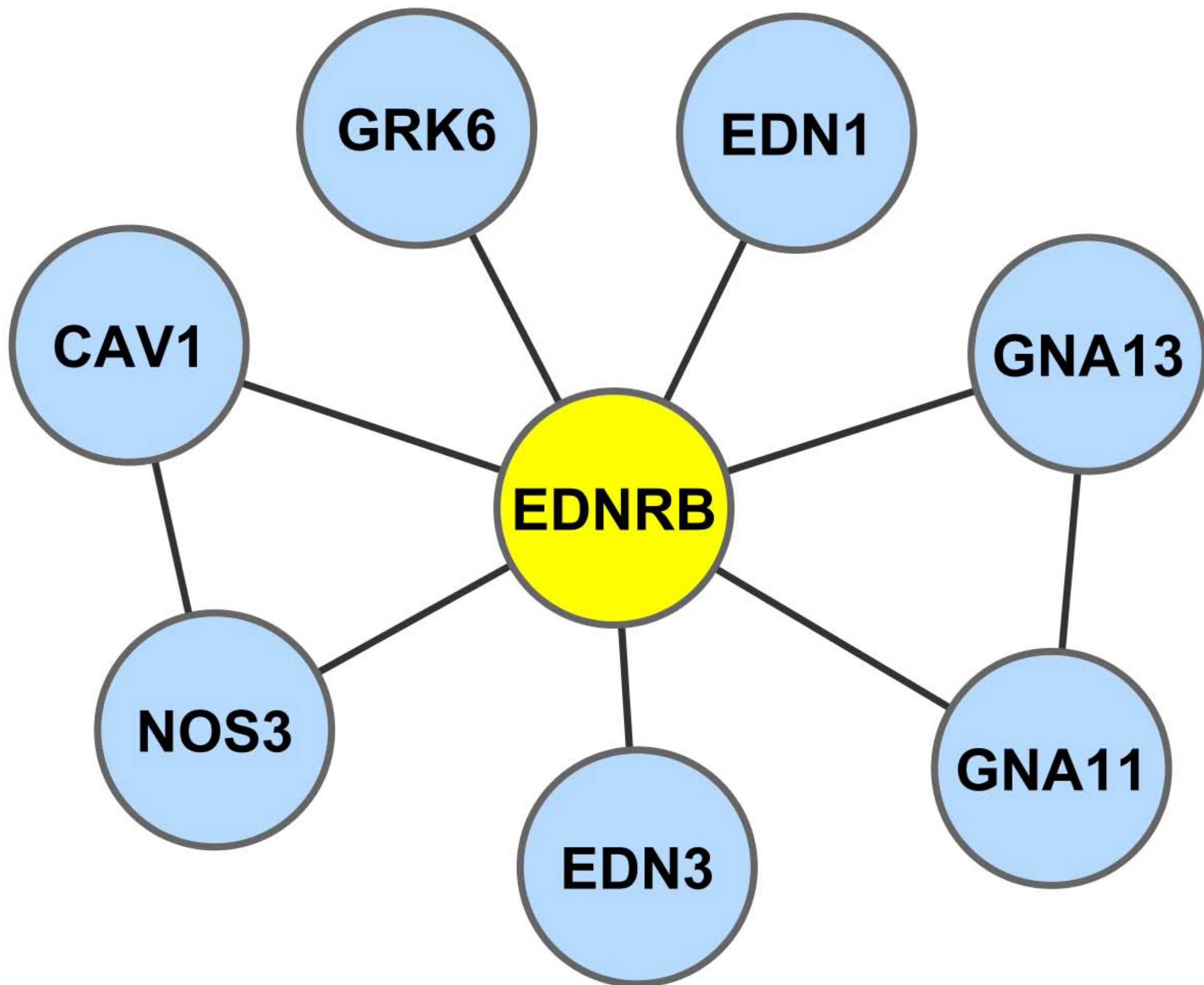

Supplement: Additional file 4 — Figure S2: EDNRB cluster obtained by considering its direct neighbors in the PPI network. Unlike the EDNRB network obtained with our GDV-based approach, this network is clearly a star-like network structure centered around EDNRB. This structure is unlikely to represent a biological pathway, since its diameter, defined as the maximum of shortest path distances between all node pairs in the network, is 2, whereas we find that about 93% of real human biological pathways from KEGG [21], when mapped onto the human PPI network, have diameters larger than 2, with the average of 6.24 and the standard deviation of 2.6. [file 1752-0509-4-84-S4.PDF]

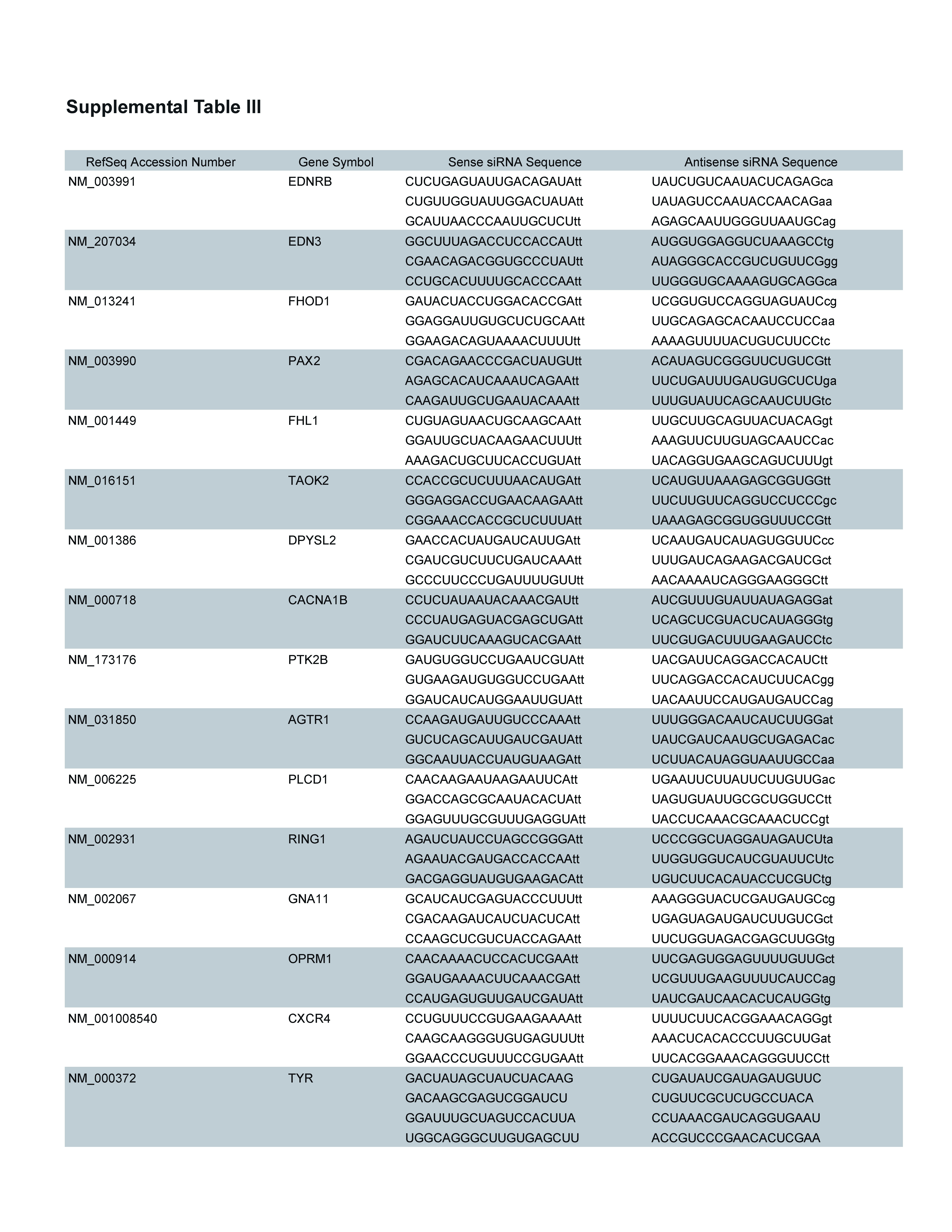

Supplement: Additional file 5 — Table S3: siRNA sequences used in this study. [file 1752-0509-4-84-S5.JPEG]

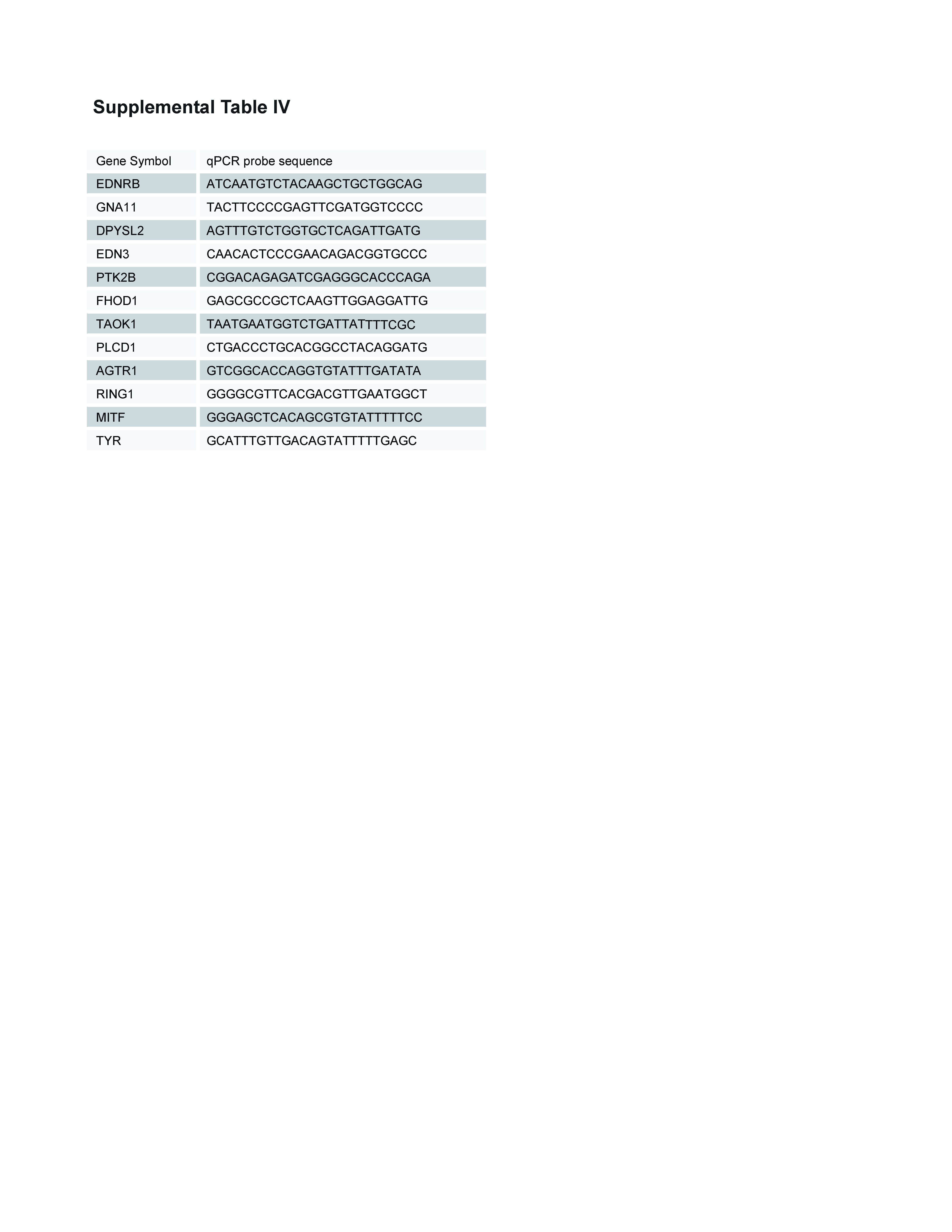

Supplement: Additional file 6 — Table S4: qPCR probe sequences used in this study. [file 1752-0509-4-84-S6.JPEG]
